# *“Here, your only relative is money…”* why slum social networks do not facilitate neighborhood community development: insights through a sanitation lens

**DOI:** 10.1186/s12889-023-17176-4

**Published:** 2023-11-25

**Authors:** Japheth Nkiriyehe Kwiringira, Joseph Rujumba, Paulino Ariho, James Mugisha, Henry Zakumumpa, Elizabeth W Perry Mohling, Mathias Akugizibwe, Innocent Kamara Tumwebaze, Charles Onyutha

**Affiliations:** 1 Department of Sociology and Population Studies, Kyambogo University, Kampala, Uganda; 2 College of Health Sciences, Makerere University, Kampala, Uganda; 3 Department of Social Work and Social Administration, Kyambogo University, Kampala, Uganda; 4 School of Public Health, Makerere University, Kampala, Uganda; 5 Center for Research on Interpersonal Violence, School of Public Health, National Safe Care Training and Research Center, Georgia State University, Georgia, USA; 6 African Population and Health Research Center, Nairobi, Kenya; 7 Department of Civil and Environmental Engineering, Kyambogo University, Kampala, Uganda

**Keywords:** Money, Slum, Social Networks, Neighborhood, Community development, Sanitation lens

## Abstract

**Introduction:**

Though social networks which are deemed vehicles of community development exist in slum areas, underdevelopment still persists in these areas. We explored the nature and role of social networks in facilitating community development in the slums of Kampala through a sanitation lens.

**Methods:**

Qualitative Social Network Analysis (SNA) was done to understand the nature of slum social networks primarily through the analysis of sanitation behavior. Data were collected through six Focus Group Discussions (FGD), six In-depth Interviews (IDIs), and 18 Key Informant Interviews (KII) with Government, civil society and private stakeholders. We used both inductive and deductive thematic analysis.

**Results:**

Four themes emerged in our analysis; i); *Unsupportive environments, uncooperative neighbours and uncertainty of tenure*: participants reported slums as unsupportive of community development due to a shortage of space, poverty and unplanned services. Tenants perceived landlords as exploitative and predatory and wished the tables are turned. This notion of cyclic exploitation did not encourage collective action for community good. Short-term economic survival trumped long-term community interests ii) *Patronage and poor service delivery*: varying degrees of patronage led to multiple forms of illegalities and violations such as tax evasion. Due to vested interests and corruption among public officials, the slum population was lethargic. iii) *Intersecting realities of poverty and unemployment*: slum dwellers lived on the margins daily. Hence, poor living conditions were a secondary concern. iv) *Social relations for personal development*: Slum social networks were driven by individual interests rather than community good. Slum dwellers prioritized connections with people of common socio-economic interests. As such social networks were instrumental only if they ‘added value’.

**Conclusion:**

Social networks in slums are only concerned about survival needs. Slums require responses that address the complexity of slum formation and broader livelihood challenges, as well as re-assessing the meaning of community. We posit that more needs to be done in understanding the meaning and workings of a sociology beyond physical societies. Poverty is a modifier of social systems and processes and should be a concern for all stakeholders involved in slum development.

## Background

 The United Nations Sustainable Development Goals (SDGs) 1 and 11 are central in tackling challenges of slums in cities across the world. Goal 1 of the SDGs is to “end poverty in all its forms everywhere” by 2030 while goal 11 target 1.1 emphasizes that, by 2030, there should be “access for all, to adequate, safe and affordable housing and basic services and upgrade slums“ [1]. The United Nations in 2010 postulated that the number of slum dwellers is projected to increase to 2 billion by 2030 and to 3 billion by 2050 if current trends of urbanization persist [2]. Achievements of goal 11 target 1.1 of the SDGs in cities by 2030 remain challenged by increasing population and poverty in slums.

Slums are places with negative implications on education, health, child wellbeing and social exclusion among others [3]. Slums also comprise an urban phenomenon which pose a major challenge to development and are likely to continue with far reaching implications on all development agenda [4]. Like in the rest of the developing world, the rapid urbanization in Uganda has been characterized by the proliferation of slums [5, 6]. Slum locations lack one or more of the following indicators: a durable and sound housing structure, access to potable water, access to improved sanitation, sufficient living space, and secure tenure [7]. These slum conditions are associated with poverty and they tend to be typified by poor housing, overcrowding, high child morbidity, high unemployment rate, and poor sanitation services including speculative development wrapped in territorial politics of informality [8–11]. There is evidence that social networks are important in striving for improved welfare and resilience in urban and rural settings [12, 13]. Furthermore, social capital is evidentially considered to be key in overcoming vulnerabilities [14, 15]. Understanding the nature and scope of existing social capital is essential to building the resilience [12] of people living slum settings [16]. However, the complexity of urban areas requires awareness of social systems and demands approaches that acknowledges existing and potential relationships [17–19]. This paper examines why; slum areas remain undeveloped notwithstanding the widespread existence of social capital in form of social networks in Kampala slums.

Social networks are sets of actors linked by social relationships or ties [20]. In most poor contexts, economic transactions take place within specific networks [21], which can be social or ethnic as well as commercial and economical [22, 23]. For the majority of the poor, social networks constitute an important, and sometimes the only means for progress [24, 25]. There is evidence that social networks reduce transaction costs between trade partners through minimizing information asymmetries between the principal and the agent as the principal knows the agent better and thus, can better anticipate his behavior [24–27]. Social networks also provide channels through which the principal can obtain ex-post information about potential misbehavior or non-compliance of the agent, thereby able to invoke sanctions on the other party [26, 28]. Through social networks and other cultural variants like social norms, there is an informal contract enforcement [24, 29]. While social norms and networks have been central in the socio-economic development discourse [30, 31], it is not clear why slums in Uganda remain undeveloped despite the widespread existence of social networks. This paper argues that slum social networks are not the same as social networks in other areas especially in non poor neighborhoods. Like in other settings, slums are characterized by norms and social networks with potential to enhance community development [32, 33]. In Uganda, the nature and role of social networks in fostering community development has not been much documented. Social networks are critical drivers of asset creation, resilience, inclusion, participation, accountability, empowerment and capabilities. The focus of this study was to present the uniqueness of slum social networks as they relate to community development from the sanitation lens.

## Methods

### Study design and setting

This was an ethnographic exploratory study conducted in Kampala city. Study participants were from three densely populated slum settlements of Jjuko, Dobbi and Gogonya; through a consultative participatory process. Study participants were invited to participate if they were eighteen (18) years of age or older and had lived in their current community for more than five years. Participants gave approval by signing the written informed consent form.

### Data collection methods

Data were collected through focus group discussions, in-depth interviews (IDIs), key informant interviews (KIIs) as well as community meetings and community windscreen /transects (see Table 1). On average, FGDs lasted 1 h, while KIIs took about 45 min.

**Table 1:** Summary of data sources

Category of participants	Number
KII (6 taken from each study slum zone)	18
IDIs (2 from each zone)	6
Community transects (This is a method where the community layout was studied by the filed team and first author)	3
FGDs (Women and men) for each zone. Discussed urban sanitation experience, prevailing sanitation situation, challenges, options, coping and future plans	6
Community meetings	2

Qualitative Social Network Analysis (SNA) [34] was done to understand the nature of slum social networks. Social network analysis has grown in its application when studying opportunities and challenges that exist in a given setting [35–38]. Qualitative SNA and other forms of urban ethnography have been found to facilitate the investigation of complex relationships [39, 40]. The agility of qualitative analysis in handling complex and dynamic networks has been previously underscored [41]. We used both inductive and deductive thematic analysis, where data were analyzed in an iterative manner [42, 43]; from reading the field notes and getting familiar with the collected data, which enabled the generation of codes as a form of preliminary analysis.

### Ethics

The study was cleared by the Institutional Review Board (IRB) at Makerere University School of Social Sciences (MAKSS REC 08.18.210) and the Uganda National Council of Science and Technology –UNCST (UNCST SS273ES). Participants gave approval by signing the written informed consent form. All methods were carried out in accordance with relevant guidelines and regulations.

## Results

The study findings are from key informant interviews (18), in-depth interviews six, three community transects and two community meetings. The study participants were resident of three densely populated slum settlements of i. Jjuko, ii. Dobbi and iii. Gogonya enlisted through a consultative participatory process. Participants were eighteen (18) years of age or older, having stayed in the area for at least five years or more. The study slums were poor urban enclaves that presented low socio-economic indices ranging from unemployment and low incomes, water, sanitation and Hygiene (WASH), poor housing, lack of access ways, poor garbage collection and congestion. We present findings along four (4) themes; *(Theme 1: Unsupportive environments, uncooperative neighbours and uncertainty of tenure; Theme 2: Patronage and poor service delivery; Theme 3: Intersecting realities of poverty and unemployment and Theme 4: Social relations for personal development).*

### Theme 1: unsupportive environments, uncooperative neighbours and uncertainty of tenure

Pervasive poverty, characterised by unplanned housing and poor sanitation were the norm in study areas. Findings indicate that a lack of space and incentives in the study slums sustained the poor living conditions. There was no incentive *to do the right things* and *to do things right*. In addition to poverty, there was indifference that was normalised, through the prevailing practices of unplanned living and a lack of regulation that were manifested in poor sanitation facilities and practices. Survival before dignity had been normalised as a form of *culture* (as patterned and repeated practices). Being a tenant was associated with no responsibility, since tenancy was seen as a transient phase. We found that tenants did not enlist long-term interests in slum areas. Property ownership was seen as the desired end. The pervasive informal and illegal practices such houses without proper sanitation, tax evasion through illegal and irregular transactions, encroaching on road reserves and public spaces including insecure tenure were seen to have negative impacts on living environments but remained unattended too. In the process, short term and personal improvement were in general, pursued as more rewarding as opposed to collective community concerns. Pursuit for short-term goals by both property owners (expanding non-regulated and uncompliant rentals) and tenants (paying as little as possible, while saving as much as possible for personal advancement) were a common reality. As a result, slum dwellers were less concerned about lasting sanitation improvements. What cut across was the slum social structure as defined by insecurity of tenure with no long-term interests in slums. Uncertainty about tenure was rationalised by the risks of displacement by both public and private actors. One resident asserted that:

*‘We do not value living in a clean environment. Why should we be bothered when we are not sure when they will destroy this place either for road works or for the rich to take over. We even do not know the real owner of this place…here we live from day to day.’* FGD, Male resident- Jjuko zone.

*‘I’m now stuck in this place and have nowhere to go! That is a reality now, I must save whatever I can to make sure I afford to stay in this place of no relatives. No one knows me here. Here your only relative is money…’* Female resident, Gogonya zone.

Tenants noted that, improvements in the sanitation status would mean increased rent and other costs and yet their incomes were not elastic. Therefore, if one needed a more sanitary and organised place, they would simply move to another place, than demanding for a better facility from the property owner. The demand for improvements in property aesthetics would be unwelcome by those that were not in a position to afford the extra cost, but also the owner since this would not attract commensurate increments in rental charges. There was consensus that places with better services and facilities are well known, but not affordable, hence, (initially) avoided. It was common for residents to move to better places when means permitted. Another practice was for landlords (current and prospective) to invest income elsewhere than investing in the upgrades and property improvements especially when tenure was insecure. There was mention of random demolitions and evictions that did not attract any compensation or rehousing. Slums also served as reserves of cheap labour where the lowly paid – low income urban poor found space in the city fabric, as these could otherwise not fit in mainstream (costly) urban life. Slums were therefore vital spaces and places in the urbanisation narrative of Uganda’s urban *Darwinism*. The survival imperative and the need to get by seemed to override other social and community considerations. There was a focus on personal concerns, and not slum community needs. Due to insecure tenure, slum dwellers had understood the slum investment ethos of minimal investment for better returns. By and large, the available self-help groups (social networks) did not consider collective action for a public good. As such, slum investments were extractive, unregulated and predatory to the slum environment.

### Theme 2: patronage and poor service delivery

There were varying degrees of patronage that led to illegalities and violations of shared goods by both patrons and their clients. Due to vested interests and corruption among public officials, the population had become lethargic viewing slums as places to be borne with. The unregulated transactions in slums such as tax evasion and unplanned land tenure; low revenue collection, lack of enforcement and a generally apathetic population that did not demand for better services sustained poor living conditions.

Slums also served as vote banks where politicians and other elites ‘*warehoused’* their voters in a complex game of political promises, political favors and dealing with their political enemies and threats. This clientelism and perverse incentives denigrated local development. Patronage in slums led to varying degrees of illegalities and planning violations, including non-compliance to public health rules and regulations. This was summed up in the following except;

*‘All those big names and politicians have their ‘army’ of voters in this place. Indirectly, many people in the slum serve the interests of those in power. There are ‘brokers’ that ‘manage’ this complexity by managing the majority for the benefit of those that gain from this chaos…’ Policy analyst/ researcher*.

In such a setting, it was reportedly not plausible to enforce public health regulations, with the very custodians of public order in these locations falling short of the very standards.

*‘We stay in a poor area, but this area makes a lot of money for the owners. If they really wanted, they would improve this place… but they are not bothered. Why should we bother either? In town, you are on your own. If you are lucky and get a friend or relative to work with, that is enough…’* In-depth interview with long stay slum dweller, Ddobi zone.

*‘Our leaders only care about themselves. When we first came here, we thought that you could share whatever was available to the community. We did not know that to defecate, you need money…. There is no service; nothing is free here. That is why we do our best to avoid paying any money. Otherwise, how do you survive? Where can you get such volumes of money? The more you stay here, the more you understand that it is up to you to survive. If you cannot, no one is bothered because we are all struggling! Female slum resident, Jjuko zone*.

Slums were devoid of safe water supply, improved sanitation, sufficient living area, durable tenure, public service aesthetics and established property rights. By and large, slum social structures have evolved as survival spaces where residents do not perceive themselves as part of the urban polity and the solution to improving slums; but improving themselves. This improvement was through eking out a living through all manner of income sources, including all round endurance and illegalities. Even in conditions of squalor and poverty, slum dwellers were aware that public interest provisions had ceased to be effective and served private gain for the urban elite. While the city had service delivery challenges, slums therein were exceptionally deprived of social services. This had served as a pointer for slum dwellers that there were no standards to be upheld and demanded. Life in the slums was to whom it may concern.

### Theme 3: intersecting realities of poverty and unemployment

Incomes in slums were low amidst limited employment opportunities. Study participants noted that most times they live from day to day without much left in terms of savings. In this case, daily income was critical. Because of this scarcity occasioned by a small capital base and trading options such as the selling of charcoal, alcohol brewing and food items on a small scale by hawking could not enable break out of poverty. In the process, slum dwellers were not bothered about bad slum sanitation and aesthetics because this was what they could afford. What was regarded as important was to meet the basic survival needs such as food and shelter. One participant asserted that;

*‘You cannot have much expectations regarding good standards when you are poor! For us the poor, we work with what is left over’* Resident-Ddobi zone.

Life in the study slums was reported to be unpredictable especially that the livelihood sources were insecure. There was a great likelihood of experiencing physical harm for children, girls and single women especially at night when they started transacting in the small trades in open air roadside markets. Even when such risk was well known, there was no alternative to taking on such risks. The risks were associated with dark unlit spaces and a lack of sanitation facilities as well as open trenches that were not addressed by local authorities. Due to the pervasive poverty, and the needed resilience, many bad situations were ignored in an effort to find space in the city. Most property owners in the studied slums were absentee landlords; therefore, they were not directly affected by the negative realities of living in slums. This disconnect further drove slum exploitation and extractive frameworks including cheap labor, poor standards in housing, water and sanitation. Slum dwellers had understood their place as *being on their own* without meaningful support from social structures that deliver public goods, social security or employment. The available social networks (economic, financial and geo-ethnic) were not concerned about the development of slums, but meeting the practical needs of income and asset acquisition; the *here and now*. This pursuit for survival among slum dwellers rendered common goods irrelevant. This was a manifestation of urban decay where those that break out of poverty as well as those with the means to improve slum conditions, do not tend to do so. Slum exploitation was seen as a means to succeed elsewhere. The challenge is how to stop this cycle. Poverty and the difficulties experienced in trying to meet basic needs had relegated dignity.

### Theme 4: social relations for personal development

The few available social networks in slums that we encountered through this study had specific targets and objectives. These target-based networks and associations focused on specific, usually income objectives for members and not the wider community. We found that these networks would not survive if their economic rationale did not exist. It is this economic rationale that was the motivation to start and join such associations and groups. Other ventures and initiatives that did not have a direct economic or capital benefit were not attractive to slum residents. Usually, slum dwellers prioritized making connections with people in common trade and interests or those that could support them to achieve the next economic status. Such relations were not usually with and not necessarily among neighbours, but rather with those carefully selected individuals for mutual benefit. Such individuals usually came together, not to discuss area progress and community development matters such as sanitation or other shared and collective services but met in savings and credit groups commonly known as Nigiina. These relations and strategies encouraged predation of shared resources with no regard for the future. There were reports of the few shared spaces having been converted to private use. This partly, in addition to the lack of planning, explains the overcrowding and haphazard structures.

Tenants argued that improved sanitation only benefitted the property owner in form of increased rental charges, and thus, as tenants, there was no incentive or motivation to support or invest in improved sanitation. The social networks and behavior types were those deemed critical for survival, while other aspects of life, like improved sanitation access, remained unattended. In order to cope with urban life that was cash driven, slum dwellers engaged in activities that initially tended to underlie survival (physiological needs, such as food and shelter). Sanitation and shared (public) services were neither an area of recurrent expenditure nor a focus of future planned expenses. In-depth discussions indicated that income earning ventures and improved sanitation (private or shared) were not aligned as shown in the voices below.

*“Under normal circumstances, people who stay and work together interact and relate very well. However, such happens in villages [and not in slum areas]. Here in slums, people mind their own business. And if they meet for business, they do not transfer the same cooperation to cleaning shared latrines”.* Local leader, Gogonya Zone.

*“Having common interest beyond income and survival groups can only work among landlords; but with tenants, it is not possible. People know that anytime they would be shifting to a different place to look for more opportunities….*” Local Council Official, Jjuko zone.

For a slum dweller, seeking the most affordable housing to escape the sun and the rain was the most important thing over and above the availability or status of sanitation. In most cases, the urban poor were at least able to escape the sun. In general, slums served as places of survival and not welfare.

Participants emphasized ‘*minding one’s’* businesses and ‘*playing it safe’* with neighbours. They argued that it was irrational, uneconomical and unsustainable to police fellow tenants around and yet everyone was struggling. This indifference did not encourage shared and public goods to flourish. The voices from FGDs illustrate this point.

*“The house is not mine, and I may leave this place anytime. Why should I be bothered? Everyone has enough troubles. What should bother me is what to eat and not where to defecate or where my neighbor defecated; properly or not.” Female FGD participant, Jjuko Zone*.

*“Here, everyone works as an individual.’ Punishing people is not easy. Even then, such people do not get ashamed. Even if you identified them, they are likely to remain the same. Because of this, proper sanitation has remained elusive.” Female tenant, Jjuko Zone*.

In the absence of the-would-be normative social structures and local systems of social control, growth and development is unlikely. Socially disjointed neighborhoods with neither community sentiment nor altruism provide scant support for structures of shared living, unless the support is provided by the market or welfare state. In this sense, slum neighborhoods were not viewed by residents as *communities*, but were viewed as mere places of residence with ‘*limited liability’*. While the notion of *community* focuses on the existence of commitment, skills, resources, and problem-solving abilities among others, this was not the case in our study setting. This understanding also underlies community capacity as being directly and indirectly influenced by the social structure and other contextual factors. We found that neighbourliness in slums was not based on social ties, but more so on resource and market systems. It was money that bridged the gaps left by the absence of functional commons (shared goods for collective gain). Slum social networks were selective, transactional in nature and focused on individual progress rather than collective progress. There was evidence that slum dwellers were members of social networks as a functional response to mitigating the harsh urban demands and not to improve the place of residence.

## Discussion

These findings indicate that the study slums represented a marginalized and fragmented subpopulation that excluded residents from mainstream society. Slum dwellers were buffeted by an array of hazardous living. While urban areas are supposed to be places of better social services, slums are unplanned, unserved and unregulated. Slum dwellers did not find advantage out of merely working for area improvements. The reduction of social interaction to interpersonal, rational and transitory exchanges left urban dwellers with networks characterized by weak and instrumental ties. There was full knowledge that slums are stepping-stones for finding better space in the city or at the very least to survive. The improvement of slums did not appear to be a priority. Insecurity of tenure, poor housing, poverty, unemployment and poor water, sanitation and hygiene services among others made the concept of a supportive community [44] elusive. To a large extent, the conventional sense of community was absent due to social exclusion, cultural fragmentation, social heterogeneity and anonymity [45], which may have influenced the lack of structured welfare [4]. From a theoretical stand point, repeated patterns are a form of culture [46, 47], as such, good sanitation not being given priority was the culture in the study slums.

While studying the provision of public goods in America and other developed democracies, Alexis de Tocqueville shows that neighbors in progressive communities were organized to address local challenges without involving authorities [48–50]. This contrasts with what we found in the slums of Kampala. There was minimal interest in community issues, potentially due to competing priorities, coupled with low capacity to produce public goods for alleviating public harm through positive externalities such as the preservation and improvement of shared spaces, clean air and conserving the environment [51].

The ability to endure was a survival instinct amidst many constraints, such as lack of employment, housing and basic public services [52] that called for many compromises [53–55], a replica of a tragedy of commons [56–59] replete with self-exploitation and unsustainable strategies. A phenomenon that warrants further investigation. Earlier studies have inferred the survival imperative among slum dwellers although less explicitly [60–62]. Collective action works best with systems than with individualistic profiles. Like Maslow’s hierarchy of needs [63], we found that slum dwellers in our study were motivated to achieve needs considered necessary for individual survival; community improvement was a lower priority.

We posit that, despite numerous material hardships [64], urban life in many ways was seen as a platform for progress, modernity and opportunity for the individual and is less often seen as a platform for societal progress. Accordingly, good sanitation was neither seen as a physiological nor a safety need. Residents in the slums prioritized looking for the most efficient way to leverage their positions in the contexts of structural deficiencies and socio-economic barriers. There is inaccuracy when analysts and scholars transform sociological conditions into psychological traits and then impute onto victims these distorted views. The culture of poverty is a case in point [65, 66]. The hierarchy of needs theory was applicable in understanding the sanitation choices in slums as *survival before dignity* and argues against the premise that poverty is a culture among the poor. The culture in slums was that people did not mind the effects of their choices on the environment. All the focus was on the likelihood of earning an income. We question the universalism of the cultural premises of the culture of poverty thesis where the poor are socialized into poverty. Rather, the urban poor who participated in our study were actively working to escape poverty.

The available slum social networks were a functional response to mitigating the harsh urban demands. It was clear from the onset that, town life was about money and not collective progress of the area. Amidst this, there was rationality in the slum [67–70] with a focus on income-earning opportunities and street survival. Such was the context of social networks and trust in slums that shaped networks, community conscience, norms and standards of practice. While reciprocity and trust [71, 72], together with shared values that emanate from the rural ‘moral’ economy, generated social capital, the case in slums was different. More than anything else, slum dwellers engaged in social networks as *bankable* ventures that were instrumental. The major issue for a slum dweller were functional and instrumental relations in the market place [73]. In our study, activities in slums were driven by private interest. Concentrated disadvantage and disorder had weakened local development leading to the dearth of distance and mobile sociologies [74–76]. While there were *no territorial communities* [77], there were networks [5, 78, 79]. We note that communities can exist without a territorial base, and indeed, territories can exist without communal ties.

### Policy and programing implications

There are studies showing that social relationships and social networks are critical factors in shaping the management of common goods. There has been evidence of shared sanitation facilities being poorly maintained that was associated with collective action failure. We propose both product and service value chains that would support and improve slum living through regular employment and regulation of shared space for the common good. Formal economic transactions come with compliance, rights and duties as well as regulations that are impersonal and tend to privilege sustainability over mere monetary gains. Urban reforms are needed in dealing with unemployment, low productivity and poor enforcement of urban planning and settlement regulations including better institutional synergies at different levels of decision making. Sustainable sanitation services are more likely to be delivered when livelihoods are secure and the capacity of local authorities to deliver services such as good sanitation is enhanced. Our findings highlight poverty as a critical modifier of social systems and processes and should be a central concern for all stakeholders involved in slum improvement interventions. At programing level, there is need for a holistic approach and deliberate efforts to address individual survival and development needs together with collective community needs. For this to happen, real collaboration is required among actors, departments and departments involved in the provision of financial, sanitation, housing and health care services to slum dwellers and those responsible for urban planning, financing and enforcement of standards.

### Strengths and limitations of the study

This was a qualitative study. As such, being non-random, it is not representative of all slums in Uganda. However, the study offers insights into slum social norms in Kampala through a sanitation framework. This provides a good starting point in better understanding life in the slums, which can be built on to work toward more sustainable urban development.

## Conclusion

Social networks in form of ties as well as innovations can be fostered and eroded by conditions and local contexts. The slum social structure of survival amidst scarcity, indifference and anonymity made it difficult for shared and common goods to thrive in the slums of Jjuko, Dobbi and Gogonya in Kampala City, Uganda. There was an embedded difficulty of having a collective action code for slum sanitation. This was because there were few options in addition to a lack of enforcement means. There were norms of free reign and networks that did not lead to the improvement of the slum. If slums offer security of tenure and a pathway to financial security, it is plausible that slum social networks can develop the community. This study places the role of social networks, urban livelihoods, poverty alleviation, good governance, social service delivery and management at the heart of community development. We call for a more comprehensive appreciation of urban poverty as a modifier of social systems and processes especially when survival is threatened. It is hoped that this realization shall help in shaping the approaches aimed at addressing poor slum sanitation and urban poverty especially the realization that actors are rational and self-aware. As such they do not just respond to regulation but are calculative in what they do on the basis of means and ends; costs and benefits. Slum realities require responses that address the complexity of slum formation and broader livelihood challenges, as well as the re-assessment of the meaning of community and fluidity to a slum dweller. Future research ought to focus on how identity, sociality and social networks can be synched to support slum greater good, change and development.

## Data Availability

All supporting data is with the first author. The data is available from the corresponding author on reasonable request.
