# Correction: *“Here, your only relative is money?”* why slum social networks do not facilitate neighborhood community development: insights through a sanitation lens

**DOI:** 10.1186/s12889-023-17360-6

**Published:** 2024-04-05

**Authors:** Japheth Nkiriyehe Kwiringira, Joseph Rujumba, Paulino Ariho, James Mugisha, Henry Zakumumpa, Elizabeth W. Perry Mohling, Mathias Akugizibwe, Innocent Kamara Tumwebaze, Charles Onyutha

**Affiliations:** 1 Department of Sociology and Population Studies, Kyambogo University, Kampala, Uganda; 2 College of Health Sciences, Makerere University, Kampala, Uganda; 3 Department of Social Work and Social Administration, Kyambogo University, Kampala, Uganda; 4 School of Public Health, Makerere University, Kampala, Uganda; 5 Center for Research on Interpersonal Violence, School of Public Health, National Safe Care Training and Research Center, Georgia State University, Georgia, USA; 6 African Population and Health Research Center, Nairobi, Kenya; 7 Department of Civil and Environmental Engineering, Kyambogo University, Kampala, Uganda

**BMC Public Health (2023) 23:2341**

10.1186/s12889-023-17176-4

The original publication of this article contained an incorrect author name. The incorrect and correct author name are shown in this correction article, the original article has been updated.

Incorrect

Liz Perry Mohlin

Correct

Elizabeth W Perry Mohling

